# The Impact of Cocaine Use and the Obesity Paradox in Patients With Heart Failure With Reduced Ejection Fraction Due to Non-ischemic Cardiomyopathy

**DOI:** 10.7759/cureus.40298

**Published:** 2023-06-12

**Authors:** Adedoyin A Akinlonu, Alvaro Alonso, Tuoyo O Mene-Afejuku, Persio Lopez, Tikal Kansara, Olatunde Ola, Savi Mushiyev, Gerald Pekler

**Affiliations:** 1 Department of Epidemiology, Emory University Rollins School of Public Health, Atlanta, USA; 2 Department of Medicine, New York Medical College, Metropolitan Hospital Center, New York, USA; 3 Hospital Medicine, Mayo Clinic Health System, La Crosse, USA

**Keywords:** obesity paradox, non-ischemic cardiomyopathy, heart failure outcomes, hospital readmission, cocaine use

## Abstract

Background

Obesity and illicit drugs are independent risk factors for developing heart failure (HF). However, recent studies have suggested that patients who already have HF and are obese have better clinical outcomes. We aim to study the effect of cocaine use on this obesity paradox phenomenon as it pertains to HF readmissions.

Methodology

In a retrospective chart analysis, we reviewed patients with a diagnosis of HF with reduced ejection fraction (HFrEF) admitted to Metropolitan Hospital in New York. We studied the association between body mass index (BMI) categories, namely, non-obese (<30 kg/m^2^) and obese (≥30 kg/m^2^), cocaine use, and the primary outcome (time to readmission for HF within 30 days after discharge). The interaction between cocaine and obesity status and its association with the primary outcome was also assessed.

Results

A total of 261 patients were identified. Non-obese status and cocaine use were associated with an increased hazard of readmission in 30 days (hazard ratio (HR) = 2.28, p = 0.049 and HR = 3.12, p = 0.004, respectively). Furthermore, cocaine users who were non-obese were over six times more likely to be re-admitted in 30 days compared to non-cocaine users who were obese (HR = 6.45, p = 0.0002).

Conclusions

Non-obese status and continued use of cocaine have a negative additive effect in impacting HF readmissions.

## Introduction

The economic burden of heart failure (HF) in the United States (US) was estimated at $20 billion in 2012, which is projected to reach about $53 billion by 2030 [1]. Much of this cost is a result of emergency department visits and hospital readmissions, with about 25% of HF patients being readmitted within one month of hospital discharge [1]. Measures to reduce the economic burden of HF, which can potentially help with curbing rising healthcare costs in the US, have become a priority for the healthcare community nationwide. The Center for Medicare and Medicaid Services (CMS), for example, has employed the Hospital Readmissions Reduction Program (HRRP), a provision of the Affordable Care Act (ACA), to prevent rehospitalizations by penalizing healthcare entities for readmissions within 30 days of hospital discharge [2]. Nonetheless, hospital readmissions decreased by only 2% two years after the inception of the HRRP in January 2012 [2]. Another initiative that could be beneficial is modifying some of the social and behavioral risk factors that predispose to HF readmissions. Obesity is considered an independent risk factor for cardiovascular disease; its age-adjusted prevalence in the US reached an estimated 40.6% in 2018 [3]. However, in persons who already have cardiovascular disease, obese or overweight status seems to be associated with a better prognosis compared to normal weight, a phenomenon labeled as the obesity paradox [4]. The use of illicit drugs such as cocaine has also been associated with poor overall cardiovascular outcomes [5]; however, there is a paucity of data on the association of cocaine use with HF outcomes after the index hospitalization. Given the rising rate of obesity and substance abuse in the US [5], an understanding of the roles these factors play in hospital readmissions among patients with HF can potentially inform a targeted public health action toward reducing rehospitalization. This article will focus mainly on the association of cocaine and obesity with outcomes in HF patients with reduced ejection fraction (HFrEF) secondary to non-ischemic cardiomyopathy (CM).

This article was previously presented as an abstract at the 23rd World Congress on Heart Disease meeting in Boston on July 29, 2018.

## Materials and methods

Study design

This was a single-center, retrospective electronic chart review and analysis of adult patients (aged ≥18 years) with newly diagnosed HFrEF treated at Metropolitan Hospital Center in New York City between January 2013 and December 2016. The protocol was approved by the Biomedical Research Alliance of New York Institutional Review Board and by the central office of the New York City Health and Hospital Corporation. A waiver of informed consent was requested and granted on the basis that this is a retrospective analysis of Metropolitan Hospital Center’s patient database, and the research meets the 45 CFR 46.116 criteria for informed consent waiver.

Patient characteristics

Patients were included in the study if they were aged 18 and above and were admitted with a diagnosis of HFrEF. HFrEF was defined as clinical HF based on the recommendations from the American College of Cardiology/American Heart Association plus left ventricular ejection fraction (LVEF) ≤40% on index transthoracic echocardiogram (TTE). Patients with ischemic cardiomyopathy based on a physician’s documentation, volume overload for any reason other than HF, and end-stage renal disease were excluded. Patients who were pregnant and those with a life expectancy of less than 30 days were also excluded from the study cohort.

Data collection

Follow-ups and evaluations are routinely performed in Metropolitan Hospital’s HF clinic. A manual review of all notes (including index hospitalization and follow-up clinic visits) for the 30-day duration of the patient’s enrollment was used to retrieve demographic information, echocardiographic measurements, as well as clinical and laboratory parameters. The predictor variables analyzed in this study were (1) active cocaine use, which was defined as either self-reported continued cocaine use, or the finding of cocaine consistently in the urine drug screen of the patient; and (2) obesity, which was defined as a body mass index (BMI) ≥30 kg/m^2^, calculated using patient’s recorded weight and height on index admission. Model parameters included age, race, sex, obesity status, cocaine use, ejection fraction range, serum creatinine and hemoglobin level, clinic follow-up, and medical comorbidities including diabetes and hypertension. The primary outcome was time to readmission for HF within 30 days after discharge, as documented in the electronic medical record. Readmission was defined as a presentation with symptoms and laboratory findings consistent with HF within 30 days after the last hospital discharge for an HF admission. Secondary outcomes were the association between BMI categories, cocaine use, and readmission for HF.

Statistical analysis

Continuous variables were expressed as mean ± standard deviation if following normal distribution or medians and interquartile range if not. Normality was assessed through normality plots. Categorical variables were expressed as frequencies and percentages. The difference between groups for continuous variables was determined using the independent Student’s t-test. The difference in categorical variables was assessed using the chi-square test. Univariate analysis to assess the unadjusted relationship between obesity and the study outcome was performed using Kaplan-Meier survival curves and log-rank testing. Survival time was calculated as the time from hospital discharge day to the date of readmission or the last day of observation. The proportional hazards assumption was assessed using log-log plots. Multivariable analyses were performed using Cox regression with stepwise selection (p < 0.15 for inclusion). The chunk test was performed to determine the effect modification by cocaine on the association between obesity and the primary outcome using the likelihood ratio to test the significance of the interaction term. All analyses were performed using SAS software, version 9.4 - 2013 (SAS Institute Inc., Cary, NC, USA).

## Results

**Figure 1:**
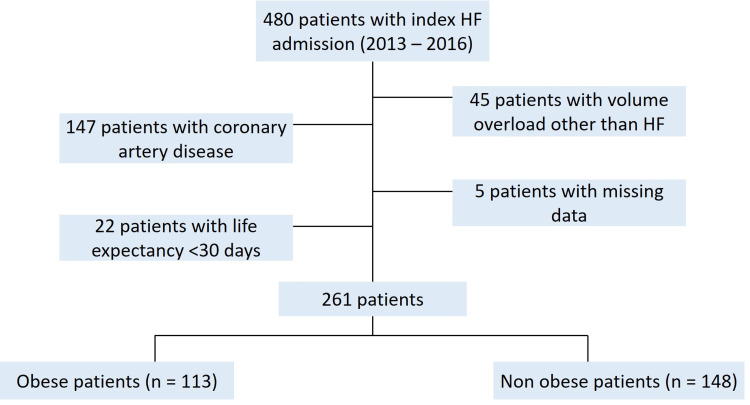
Cohort selection details. HF = heart failure

A total of 480 patients with HFrEF were identified, and 219 were excluded due to volume overload for reasons other than HF, a history of coronary artery disease, a life expectancy of fewer than 30 days, or missing data, leaving 261 subjects (see Figure 1). The clinical characteristics of the study subjects are summarized in Table 1.

**Table 1:** Baseline characteristics of patients by obesity status as defined by BMI. Asterisks indicate statistical significance. AA = African American; BMI = body mass index; BP = blood pressure; SD = standard deviation

Variable	All patients (n = 261)	Obese (n = 113)	Non-obese (n = 148)	P-value
Age (±SD), years	59 (±12.6)	56.3 (±11.6)	61 (±13)	0.002*
Male gender	204 (78.2%)	81 (71.7%)	123 (83.1%)	0.03*
Race				0.02*
AA	128 (49.1%)	62 (54.9%)	66 (44.6%)	
Hispanic	89 (34.1%)	27 (23.9%)	62 (41.9%)	
White	10 (3.8%)	6 (5.3%)	4 (2.7%)	
Others	34 (13%)	18 (15.9%)	16 (10.8%)	
BMI (±SD)	30 (±7.9)	37.2 (±6.5)	24.5 (±3.0)	<0.001*
Diabetes	93 (35.6%)	53 (46.9%)	40 (27%)	0.001*
Length of stay (±SD)	4.6 (±4)	4.9 (±4.5)	4.5 (±3.8)	0.42
One-week follow-up	58 (22.2%)	24 (21.2%)	34 (23%)	0.76
Systolic BP (±SD)	144 (±29)	145 (±30)	143 (±27)	0.61
Ejection Fraction (%)				0.03*
<25	142 (54%)	56 (49.6%)	86 (58.1%)	
25–34	81 (31.7%)	33 (29.2%)	48 (32.4%)	
35–40	38 (14.3%)	24 (21.2%)	14 (9.5%)	
Serum creatinine (±SD)	1.42 (±0.95),	1.43 (±0.75)	1.42 (±1.08)	0.93
Hemoglobin (±SD)	12.4 (±2.05)	12.2 (±2.1)	12.4 (±2.0)	0.37
Cocaine use	50 (19.2%)	21 (18.6%)	29 (19.6%)	0.88

The mean age of our population was 59 ± 12.6 years, and about 78% of the patients were male (Table 1). The prevalence of obesity was 43%. Thirty-two (12.3%) patients were readmitted within 30 days. Patients who were readmitted had a significantly lower BMI than those who were not readmitted (26 ± 5.0 vs 31 ± 8.1; p = 0.002). For the entire study cohort, 19.2% were cocaine users. The number of cocaine users did not differ significantly between the obese and non-obese groups.

**Figure 2:**
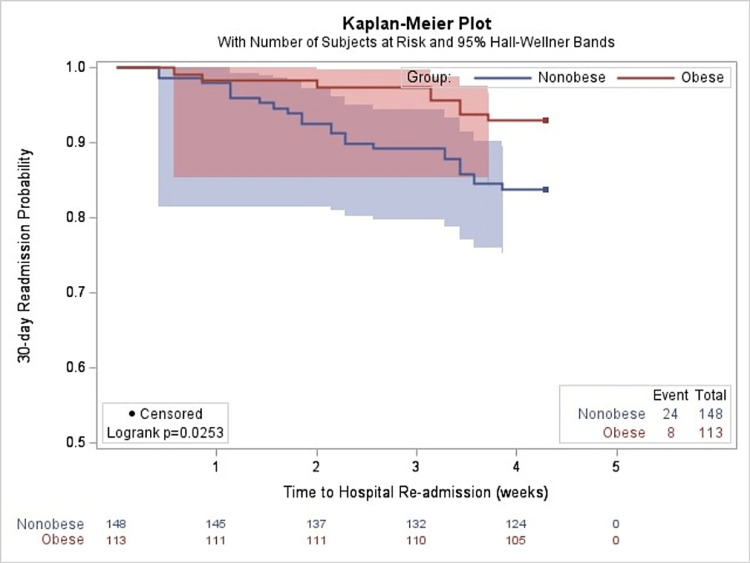
Kaplan-Meier probability estimates of 30-day readmission rates for obese versus non-obese individuals.

**Figure 3:**
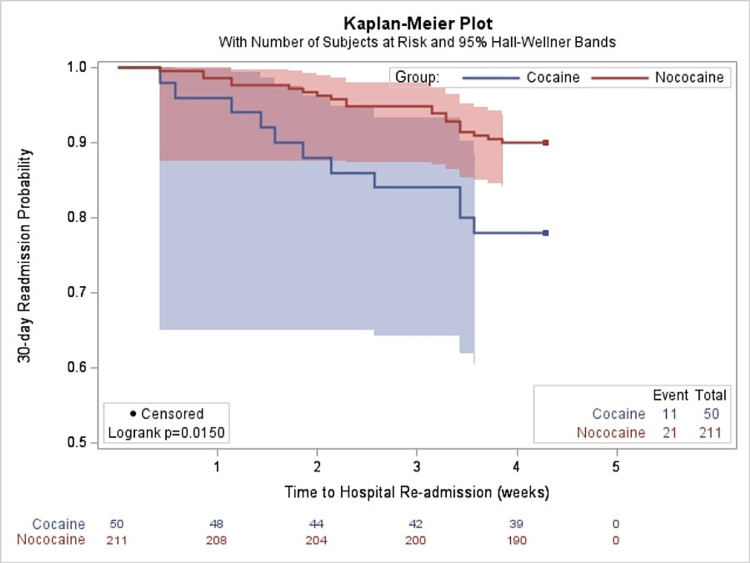
Kaplan-Meier probability estimates of 30-day readmission rates for cocaine versus non-cocaine users.

Univariate analysis showed that the hazard for 30-day hospital readmission for non-obese patients was 2.4 times the hazard for obese patients (95% confidence interval (CI) = 1.09-5.38). Moreover, cocaine users were 2.4 times more likely to be readmitted compared to non-cocaine users (95% CI = 1.16-4.97). Kaplan-Meier survival estimates comparing obese and non-obese patients revealed that obesity was associated with a reduced risk of hospital readmission for HF after hospital discharge (p = 0.025) (Figure 2). A separate Kaplan-Meier survival curve comparing cocaine versus non-cocaine users showed an increased risk of HF readmission among cocaine users (p = 0.015) (Figure 3).

We further assessed these findings through multivariable analysis by Cox regression with stepwise selection of model parameters. The stepwise selection procedure resulted in the inclusion of the following covariates in the multivariable model: obesity status, cocaine use, age, length of stay, systolic blood pressure, and hemoglobin. As no significant interaction was found between obesity and cocaine use by the likelihood ratio test, the interaction parameter was dropped, and a reduced model was used. After adjustment, obesity and a non-cocaine use status remained beneficial for HF prognosis in our study. In particular, the hazard for hospital readmission for subjects in the non-obese group was more than double the hazard for those in the obese group (hazard ratio (HR) = 2.28, CI: 1.03-5.20) (Table 2).

**Table 2:** Cox regression likelihood estimates of 30-day readmission for HF. HF = heart failure; LOS = length of stay; SBP = systolic blood pressure; Hb = hemoglobin

Parameter	HR	95% CI	P-value
Non-obese vs. obese status	2.28	1.03–5.20	0.049
Cocaine vs. no cocaine use	3.12	1.42–6.86	0.005
Age (per 10 years)	1.30	0.98–1.84	0.07
LOS (per day)	0.86	0.73–1.00	0.06
SBP10 (per 10 mmHg)	0.86	0.75–0.97	0.02
Hb (per g/dL)	0.82	0.71–0.98	0.025

**Figure 4:**
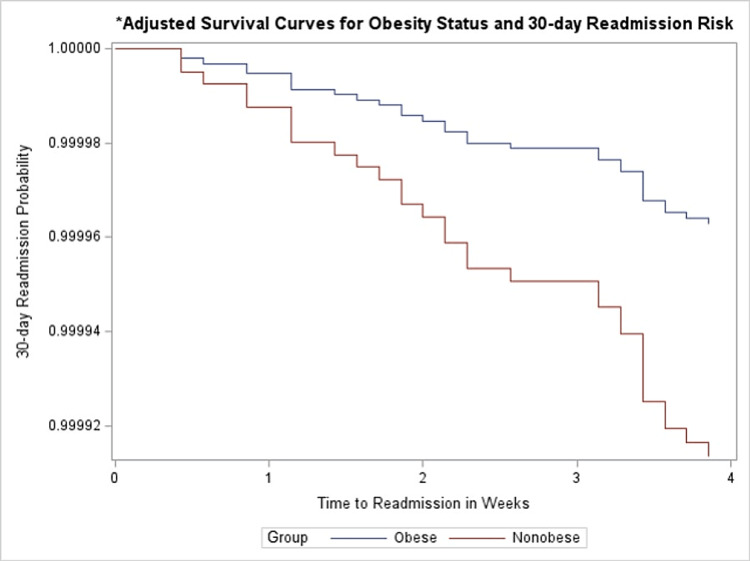
Adjusted curve of the association between obese status and primary outcome. * = Adjusted for age, length of stay, systolic blood pressure, and hemoglobin.

The adjusted survival curve supports this finding with non-obese subjects having a shorter time to readmission compared to obese patients (Figure 4). The deleterious effect of cocaine use remained after adjustment for covariates (HR = 3.12, 95% CI = 1.42-6.86) (Table 2). The results also revealed that for every 1 g/dL increase in hemoglobin, the likelihood of readmission was reduced by 18% (HR = 0.82, 95% CI: 0.71-0.98). Furthermore, for every 10 mmHg increase in systolic blood pressure, the hazard of rehospitalization was reduced by 14% (HR = 0.86, 95% CI: 0.75-0.97). Although ejection fraction and diabetes had significant between-group differences (Table 1), they were not included in the final model during the stepwise selection of parameters for multivariate analysis. We found no statistically significant effect of increasing age on the hazard of hospital readmission.

**Figure 5:**
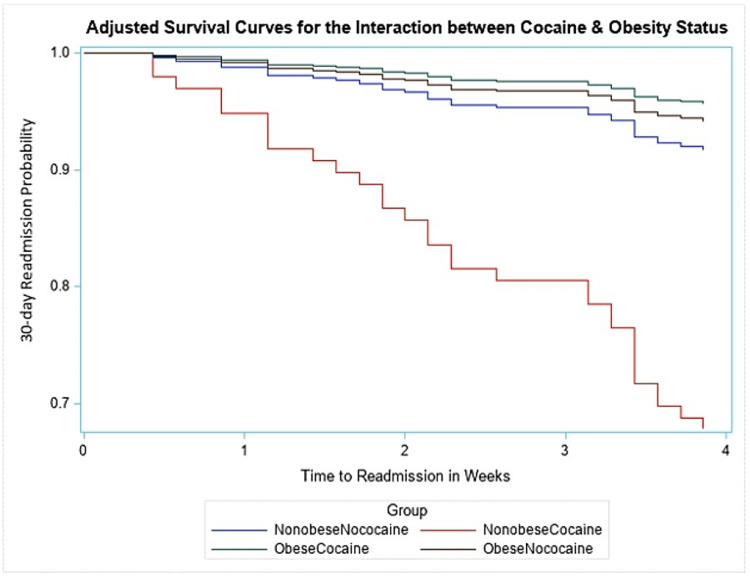
Adjusted curve of the interaction of obese status with cocaine and the primary outcome.

A subanalysis of the combined effect of cocaine use and obese status on 30-day readmission was performed by dividing the subjects into four categories, namely, non-obese/cocaine (group 1), non-obese/no cocaine (group 2), obese/cocaine (group 3), obese/no cocaine (group 4) (Table 3). Subjects in group 4 were used as the reference category for the Cox regression subgroup analysis, adjusting for covariates in the model. We found that subjects who were in the non-obese/cocaine group were more than six times as likely to be readmitted in 30 days after hospital discharge compared to subjects in the no cocaine/obese group (HR = 6.45, 95% CI = 2.39-17.4) (Table 3). This was also reflected in the adjusted survival curve that explored the interaction between the two variables (Figure 5).

**Table 3:** Cox regression estimates of 30-day readmission combining cocaine and obesity status. Adjusted for age, length of stay, systolic blood pressure, and hemoglobin.

Parameter	HR	95% CI
Obese/No cocaine	Reference	-
Non-obese/Cocaine	6.45	2.3–17.41
Non-obese/No cocaine	1.44	0.57–3.64
Obese/Cocaine	0.74	0.09–6.24

## Discussion

Despite advancements in medical diagnostics and therapeutics for HF, rehospitalization remains a challenge across health systems in the US. An estimated 25% of Medicare patients will have an HF readmission within 30 days of discharge from HF hospitalization [2,6]. While poor inpatient care, especially in low-resource hospitals, is a major contributing factor to rehospitalization, a myriad of other external factors such as socio-behavioral influences need to be considered. Exacerbations of HF that lead to readmission can result when people engage in high-risk behaviors such as eating unhealthy diets, physical inactivity, and abusing illicit drugs.

In the US, more than 30% of adults do not engage in physical activity, and about two-thirds are either overweight or obese [3,7]. The association between obesity and HF survival has been well studied, though its implications remain very contentious [8,9]. A study by Mandviwala et al. did find a protective effect of obesity on mortality among patients with HF with preserved ejection fraction, but the opposite was the case with hospital readmission, as the authors reported that an increasing BMI independently increased the risk of HF rehospitalizations [10]. Similarly, a study by Zamora et al. on obesity status and HF outcomes showed mixed results. They found a non-statistically significant association between obesity status and HF readmission among patients with ischemic CM across the BMI spectrum, while the obesity paradox was significantly demonstrated among the cohort with non-ischemic CM [11]. The latter is consistent with the findings of our study which focused on patients with non-ischemic CM. The varied conclusions from several studies of the effect of obesity on HF outcome suggest that the etiology of HF might be a key factor in determining how and when the obesity paradox phenomenon can be applied [11].

In view of the increase in the number of illicit drug users in the US in recent years [12,13], studies that explore the varying cardiotoxic effect of cocaine to educate the public are imperative. While a vast literature on the association between cocaine use and cardiovascular diseases exists, studies on the prognostic implication of cocaine use specifically on patients known to have HF is scarce [14]. Our study found cocaine use to be an independent predictor of HF readmissions, an association that was expected given prior reports of cocaine’s adverse effect on general cardiovascular outcomes [15]. Cocaine’s deleterious effect on the heart is known to be enhanced by the presence of other toxic drugs [16]; however, we found no study that has investigated the interaction of cocaine with other research-proven predictors of cardiovascular outcomes. Though there was no modifying or confounding effect of cocaine use on the obesity paradox phenomenon, our analysis revealed that patients who used cocaine and were non-obese had a higher rate of hospital readmission compared to patients who were obese and did not use cocaine. This finding implies the possibility of a negative additive effect of cocaine use and a non-obese status on HF outcomes.

It was not surprising that we found a reduced likelihood of hospital readmission with increasing hemoglobin levels. Anemia in HF patients is multifactorial and can result from functional iron deficiency due to chronic inflammation, low erythropoietin production that occurs from the cardio-renal syndrome, and a tendency for bone marrow unresponsiveness [17,18]. The resulting low hemoglobin leads to a significantly reduced oxygen-carrying capacity of the blood, putting undue pressure on the heart to increase cardiac output. This can worsen the function of an already compromised left ventricle [17,18]. Several studies have corroborated this association [19-21]. The probability of HF hospitalization and mortality was found to be higher among HF patients with anemia compared to those without anemia across the HF spectrum in a large Swedish HF registry study [21]. Patients who are hypertensive are more tolerable to guideline-directed medical therapy (GDMT) than patients who have low blood pressure [22]. Thus, patients with high blood pressure are likely to adhere to GDMT resulting in less likelihood of hospital readmission.

We will like to acknowledge the limitations associated with this study. This is a retrospective chart review, which limits the available information regarding adherence to GDMT, frequency and route of cocaine use, and pre-HFrEF BMI. The diagnosis of non-ischemic CM was identified based on administrative data and physician documentation, with limited data for retrospective adjudication. Social determinants of health such as economic condition, educational attainment, and marital status could also not be ascertained given the retrospective nature of the study. Moreover, anthropometric parameters used for analysis were those recorded on admission, and thus we assumed there were no changes in the value of these parameters during admission. In addition, the use of other anthropometric measures of obesity such as waist/hip ratio may yield a different outcome than using BMI. As the study was limited to just one of the several New York City hospital corporation facilities, we could not ascertain if patients were readmitted to the other health system facilities. Also, the death of a patient before readmission is a competing risk that was not adjusted for in this study. With the sample size being small, we were restricted in the number of covariates that could be included in the regression model. Thus, stepwise selection had to be used to ensure we are adjusting for the strongest confounders. Finally, because it is not a randomized study, we are not able to draw definitive conclusions regarding the association between variables.

## Conclusions

In summary, a low BMI is independently associated with a higher likelihood of 30-day hospital readmission among patients with HFrEF. This beneficial effect of a high BMI in reducing readmissions after an HF-related hospitalization is not altered by active cocaine use. Furthermore, continued cocaine use potentiates the effect of a low BMI on the risk of readmission after an acute HF hospitalization. Treatment of patients with anemia and hypotension should be optimized before hospital discharge. Further multicenter studies are needed to corroborate these findings.
